# Effects of bone marrow mononuclear cells from healthy or ovalbumin-induced lung inflammation donors on recipient allergic asthma mice

**DOI:** 10.1186/scrt496

**Published:** 2014-09-09

**Authors:** Soraia C Abreu, Mariana A Antunes, Lucas Mendonça, Vivian C Branco, Elga Bandeira de Melo, Priscilla C Olsen, Bruno L Diaz, Daniel J Weiss, Bruno D Paredes, Debora G Xisto, Marcelo M Morales, Patricia RM Rocco

**Affiliations:** Laboratory of Pulmonary Investigation, Carlos Chagas Filho Institute of Biophysics, Federal University of Rio de Janeiro, Rio de Janeiro, RJ Brazil; Laboratory of Cellular and Molecular Physiology, Carlos Chagas Filho Institute of Biophysics, Federal University of Rio de Janeiro, Rio de Janeiro, RJ Brazil; Laboratory of Clinical Bacteriology and Immunology, School of Pharmacy, Federal University of Rio de Janeiro, Rio de Janeiro, RJ 21941-902 Brazil; Laboratory of Inflammation, Carlos Chagas Filho Institute of Biophysics, Federal University of Rio de Janeiro, Rio de Janeiro, RJ Brazil; Department of Medicine, University of Vermont, College of Medicine, 226 Health Sciences Research Facility, Burlington, Vermont 05405 USA; Laboratory of Cellular and Molecular Cardiology, Carlos Chagas Filho Institute of Biophysics, Federal University of Rio de Janeiro, Rio de Janeiro, RJ Brazil

## Abstract

**Introduction:**

Asthma is characterized by a chronic inflammatory process which may lead to several changes in bone marrow cell composition. We hypothesized that bone marrow mononuclear cells (BMMCs) obtained from ovalbumin (OVA)-induced lung inflammation mice may promote different effects compared to BMMCs from healthy donors in a model of allergic asthma.

**Methods:**

C57BL/6 mice were randomly assigned to two groups. In the OVA group, mice were sensitized and challenged with ovalbumin, while healthy animals (control group) received saline using the same protocol. BMMCs were analyzed by flow cytometry 24 hours after the last challenge. After BMMC characterization, another group of OVA mice were further randomized into three subgroups to receive intratracheal saline (BMMC-SAL), BMMCs from control or BMMCs from OVA mice (BMMC-Control and BMMC-OVA, respectively; 2x10^6^ cells/mouse), 24 hours after the last challenge.

**Results:**

BMMC-OVA exhibited an increased percentage of eosinophils, monocytes and hematopoietic precursors, while mesenchymal stem cells decreased, as compared with BMMC-Control. BMMCs from both donor groups reduced airway resistance, alveolar collapse, bronchoconstriction index, eosinophil infiltration, collagen fiber content in alveolar septa and levels of interleukin (IL)-4, IL-5, IL-13, interferon-γ, transforming growth factor-β, and vascular endothelial growth factor in lung homogenates. However, the benefits of BMMCs were significantly more pronounced when cells were obtained from control donors.

**Conclusion:**

Both BMMC-Control and BMMC-OVA reduced the inflammatory and remodeling processes; nevertheless, BMMC-Control led to a greater improvement in lung morphofunction, which may be due to different BMMC composition and/or properties.

## Introduction

Asthma is a chronic inflammatory disease characterized by airflow obstruction and airway hyperresponsiveness arising from airway inflammation and remodeling [1]. The imbalance between tissue injury and repair caused by chronic inflammation leads to several structural changes that characterize the remodeling process of asthma. These changes include subepithelial fibrosis, collagen and elastic fiber deposition, goblet cell and smooth muscle cell hyperplasia and hypertrophy, vascular proliferation, and extracellular matrix abnormalities [2]. The changes play a key role in the pathophysiology of asthma and are associated with persistent decline in pulmonary function [3] despite treatment with inhaled corticosteroids [4]. New strategies that hasten the repair process and attenuate inflammation and remodeling are therefore required.

Systemic or direct airway administration of cell populations including mesenchymal stem (stromal) cells (MSCs), derived from bone marrow [5–8] or other sources, or a heterogeneous population of bone marrow mononuclear cells (BMMCs) [9, 10] provides a potential new therapeutic approach for chronic inflammation in asthma. In particular, administration of MSCs or BMMCs during either antigen sensitization or challenge can decrease lung inflammation and airway hyperresponsiveness [9, 11]. Notably, a recent study from our group reported that BMMCs from healthy animals were associated with greater benefits in terms of reducing levels of fibrogenesis-related growth factors compared with MSCs, yielding better lung function [11]. As BMMCs can be administered easily and safely on the same day as harvesting, at lower costs, and without risk of the reaction to allogeneic non-HLA matched cells potentially provoked by MSC administration, the idea of treating asthma using autologous BMMCs is attractive [12]. However, the bone marrow microenvironment may be altered by the chronic inflammatory process of asthma, changing the composition and properties of BMMCs.

We therefore hypothesized that: BMMC composition would differ in mice with ovalbumin (OVA)-induced lung inflammation; and when administered for therapeutic purposes, BMMCs obtained from OVA-induced lung inflammation mice would promote different effects compared with BMMCs from healthy donors in a model of allergic asthma. For this purpose, a comparative assessment of BMMCs from healthy and OVA-induced lung inflammation mice was conducted by flow cytometry. Subsequently, lung mechanics and histology, airway responsiveness, collagen fiber content in airways and alveolar septa, and expression of cytokines and growth factors were comparatively assessed following intratracheal administration of saline, BMMCs from healthy mice, and BMMCs from OVA-induced lung inflammation mice.

## Methods

This study was approved by the Ethics Committee of the Health Sciences Centre, Federal University of Rio de Janeiro, Brazil (CEUA-019). All animals received humane care in compliance with the ‘Principles of Laboratory Animal Care’ formulated by the National Society for Medical Research and the ‘Guide for the Care and Use of Laboratory Animals’ prepared by the Institute of Laboratory Animal Resources and published by the National Institutes of Health (NIH Publication No. 86-23, revised 1996).

### Animal preparation and experimental allergic lung inflammation protocol

One hundred female C57BL/6 mice (weight 22.5 ± 2.5 g, aged 2 months) were used. Twelve mice were used for extraction and characterization of BMMCs. Thirty-two mice were used for assessment of airway responsiveness after methacholine challenge (*n* = 8/group). As methacholine and the bronchoalveolar lavage technique may affect lung histological analysis, another 28 mice were used to evaluate lung mechanics and histology as well as levels of cytokines and growth factors (*n* = 7/group), whereas 28 animals were used for analysis of total and differential cell counts in bronchoalveolar lavage fluid (BALF) (*n* = 7/group).

**Figure 1:**
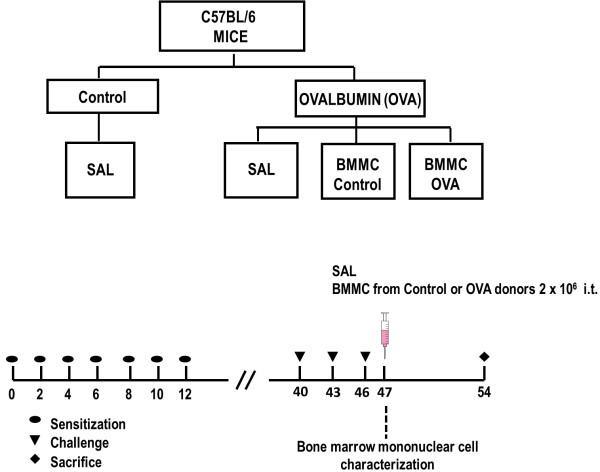
**Flowchart and timeline of the study design.** Control, mice sensitized and challenged with saline and treated with saline; OVA, mice sensitized and challenged with ovalbumin; OVA-SAL, mice sensitized and challenged with ovalbumin and treated with saline; OVA-BMMC-Control, mice sensitized and challenged with ovalbumin and treated with bone marrow mononuclear cells (BMMCs) obtained from control donors, 24 hours after last challenge (2 × 10^6^ cells/mouse); OVA-BMMC-OVA, mice sensitized and challenged with ovalbumin and treated with BMMCs obtained from OVA donors, 24 hours after last challenge (2 × 10^6^ cells/mouse). All data were analyzed on day 54.

All animals were randomly assigned to two groups. In the OVA group, mice were immunized using an adjuvant-free protocol by intraperitoneal injection of sterile OVA (10 μg OVA in 100 μl saline) on 7 alternate days (Figure 1). Forty days after the beginning of sensitization, 20 μg OVA in 20 μl saline were instilled intratracheally. This procedure was performed three times with 3-day intervals between applications [13]. In healthy animals (control group), sterile saline solution was administered using the same protocol. Another group of OVA mice was further randomized into three subgroups to receive, via intratracheal instillation, sterile saline solution (0.9% NaCl, 50 μl; OVA-SAL group) or BMMCs from control or OVA-induced lung inflammation donors (BMMC-Control and BMMC-OVA group, respectively; 2 × 10^6^ cells/mouse), 24 hours after the last intratracheal challenge (Figure 1).

### Extraction, isolation, and characterization of bone marrow mononuclear cells

Twenty-four hours after the last saline or OVA challenge, BMMCs were extracted from adult mice (two mice pooled (two femurs and two tibias) from three independent experiments), characterized by flow cytometry, and administered on the day of collection. Briefly, under anesthesia with intravenous ketamine (25 mg/kg) and xylazine (2 mg/kg), animals were euthanized and bone marrow cells were aspirated from the femur and tibia by flushing the bone marrow cavity with Dulbecco’s modified Eagle’s medium (Life Technologies, Grand Island, NY, USA). After a homogeneous cell suspension was achieved, cells were centrifuged (400 × *g* for 10 minutes), resuspended in Dulbecco’s modified Eagle’s medium, added to Ficoll-Hypaque (Histopaque 1083; Sigma Chemical Co., St. Louis, MO, USA), and again centrifuged, and the mononuclear cell fraction was washed with phosphate-buffered saline (PBS). Cells were counted in a Neubauer chamber with Trypan Blue for evaluation of viability. For the administration of saline or BMMCs, mice were anesthetized with sevoflurane, the trachea of each mouse exposed by ventral neck dissection, and 2 × 10^6^ cells were slowly injected by transtracheal puncture.

### Flow cytometry immunophenotyping

BMMCs were obtained from six mice in each group. Mice were pooled in pairs to achieve 1 × 10^7^ bone marrow cells and initiate mononuclear cell isolation. BMMCs were isolated by Ficoll-Hypaque density gradient centrifugation and their subpopulations were characterized by flow cytometry using the following commercially available specific surface monoclonal antibodies: anti-mouse Gr-1 (fluorescein isothiocyanate), anti-mouse SiglecF (phycoerythrin), anti-mouse CD45 (phycoerythrin), anti-mouse CD11b (PECy5), anti-mouse CD34 (AlexaFluor 647), anti-mouse CD3 (PECy5), anti-mouse CD4 (fluorescein isothiocyanate), anti-mouse CD19 (PECy5), and anti-mouse CD73 (Alexa Fluor 647) (BD Biosciences, San Diego, CA, USA). Antibody panels were used to detect eosinophils (Gr-1^+^SigleF^+^), monocytes (CD45^+^/CD11b^+^), hematopoietic precursors (CD45^–^CD11b^–^CD34^+^), T-helper lymphocytes (CD45^+^/CD3^+^/CD4^+^), B lymphocytes (CD19^+^), and MSCs (CD45^–^/CD11b^–^/CD73^+^). Data were acquired with a BD FACS Calibur cytometer (Becton Dickinson, Mansfield, MA, USA) by collecting 10,000 events using CellQuest software (BD Biosciences). Analysis of data was performed using FlowJo software v.7.6.5 (Treestar, Inc., San Carlos, CA, USA).

### Mechanical parameters

One week after cell therapy, the animals were sedated (diazepam 1 mg/kg intraperitoneally), anesthetized (thiopental sodium 20 mg/kg intraperitoneally), tracheotomized, paralyzed (vecuronium bromide, 0.005 mg/kg intravenously), and ventilated with a constant flow ventilator (Samay VR15; Universidad de la Republica, Montevideo, Uruguay) set to the following parameters: frequency, 100 breaths/minute; tidal volume, 0.2 ml; and fraction of inspired oxygen, 0.21. The anterior chest wall was surgically removed and a positive end-expiratory pressure of 2 cmH_2_O was applied. Airflow and tracheal pressure were measured [14]. Lung mechanics were analyzed by the end-inflation occlusion method [15]. In an open chest preparation, tracheal pressure reflects transpulmonary pressure. Briefly, after end-inspiratory occlusion, there is an initial fast drop in transpulmonary pressure (ΔP_1,L_) from the preocclusion value down to an inflection point, followed by a slow pressure decay (ΔP_2,L_), until a plateau is reached. This plateau corresponds to the elastic recoil pressure of the lung. ΔP_1,L_ selectively reflects the pressure used to overcome the airway resistance. ΔP_2_,_L_ reproduces the pressure spent by stress relaxation, or viscoelastic properties of the lung, together with a small contribution of pendeluft. Static lung elastance was determined by dividing the elastic recoil pressure of the lung by the tidal volume. Lung mechanics measurements were performed 10 times in each animal. All data were analyzed using ANADAT data analysis software (RHT-InfoData, Inc., Montreal, QC, Canada).

### Airway responsiveness

One week after cell therapy, mice were sedated (diazepam 1 mg/kg intraperitoneally), anesthetized (thiopental sodium 20 mg/kg intraperitoneally), tracheostomized, and paralyzed (vecuronium bromide, 0.005 mg/kg intravenously) for pulmonary function and hyperreactivity assessment in a FinePoint R/C Buxco Platform (Buxco Electronics, Sharon, CT, USA). Airflow and transpulmonary pressure were recorded using a Buxco Pulmonary Mechanics Processing System (Buxco Electronics, Wilmington, NC, USA), which was used to calculate airway resistance. Analog signals from the computer were digitized using a Buxco analog-to-digital converter (Buxco Electronics). Mice were stabilized for 5 minutes, and increasing methacholine concentrations (3, 9, and 27 mg/ml; Sigma Chemical Co.) were aerosolized for 5 minutes each. Baseline resistance was assessed with aerosolized PBS. The results were expressed as the mean absolute values of airway resistance responses recorded during 5 minutes after the administration of aerosolized methacholine [16].

### Lung histology

Laparotomy was performed immediately after the determination of lung mechanics and heparin (1,000 IU) was injected into the vena cava. The trachea was clamped at end expiration, and the abdominal aorta and vena cava were sectioned, producing a massive hemorrhage that quickly killed the animals. The left lung was flash-frozen by immersion in liquid nitrogen, fixed with Carnoy solution, and embedded in paraffin [17]. Slices (4 μm thick) were cut, deparaffinized, and stained with hematoxylin and eosin.

Lung histology analysis was performed using an integrating eyepiece with a coherent system consisting of a grid with 100 points and 50 lines of known length coupled to a conventional light microscope (Olympus BX51; Olympus Latin America Inc., São Paulo, SP, Brazil). The volume fraction of collapsed and the normal pulmonary areas, magnitude of bronchoconstriction, and number of mononuclear and polymorphonuclear cells in pulmonary tissue were determined by the point-counting technique [18, 19] across 10 random, noncoincident microscopic fields [14, 20, 21]. Collagen fibers (Picrosirius polarization method) were quantified in airways and alveolar septa using Image-Pro Plus 6.0 software [13, 22].

### Bronchoalveolar lavage fluid

To evaluate BALF, a polyethylene cannula was inserted into the trachea and a total volume of 1.0 ml PBS containing 10 mM ethylenediamine tetraacetic acid was instilled and aspirated. Samples were centrifuged at 300 × *g* for 10 minutes. The supernatant was removed and the pellet resuspended in 0.25 ml PBS. Total leukocyte counts in BALF were performed in Neubauer chambers under light microscopy after dilution of the samples in Türk solution (2% acetic acid). Differential leukocyte counts were performed in cytocentrifuged smears stained by the May–Grünwald–Giemsa method.

### Enzyme-linked immunosorbent assay

Levels of interleukin (IL)-4, IL-13, vascular endothelial growth factor (VEGF), interferon gamma (IFNγ; PeproTech, Rocky Hill, NJ, USA), and transforming growth factor beta (TGFβ; R&D, Minneapolis, MN, USA) were quantified in lung tissue by enzyme-linked immunosorbent assay as described by the manufacturer. Results are expressed as picograms per microliter.

### Statistical analysis

Student’s *t* test was used to compare flow cytometry data. The other variables were compared using one-way analysis of variance followed by Tukey’s test. Nonparametric data were analyzed using the Kruskal–Wallis test followed by Dunn’s test. Parametric data were expressed as mean ± standard deviation, while nonparametric data were expressed as median (interquartile range). All tests were performed using the SigmaStat 3.1 statistical software package (Jandel Corporation, San Raphael, CA, USA), and statistical significance was established as *P* < 0.05.

## Results

### Bone marrow mononuclear cells were affected by ovalbumin-induced lung inflammation

**Figure 2:**
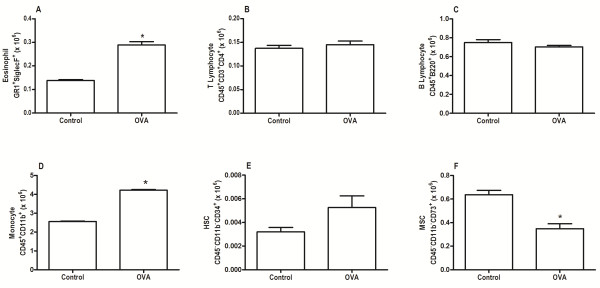
**Eosinophils, T-helper lymphocytes, B lymphocytes, monocytes, hematopoietic stem cells, and mesenchymal stromal cells.** Percentages of **(A)** eosinophils, **(B)** T-helper lymphocytes, **(C)** B lymphocytes, **(D)** monocytes, **(E)** hematopoietic stem cells (HSCs), and **(F)** mesenchymal stem (stromal) cells (MSCs) in the bone marrow mononuclear cell populations. Control, mice sensitized and challenged with saline; OVA, mice sensitized and challenged with ovalbumin. *Significantly different from control (*P* < 0.05; Student's *t* test).

The bone marrow mononuclear fraction of OVA mice exhibited an increase in the percentage of eosinophil, monocyte, and hematopoietic stem cell populations, as well as a reduction in MSC populations, when compared with bone marrow mononuclear fraction of control mice (Figure 2). No significant differences in B lymphocytes and T lymphocytes were observed in BMMC-OVA mice compared with BMMC-Control mice.

### Bone marrow mononuclear cells improved lung mechanics and airway responsiveness regardless of BMMC donor group

**Figure 3:**
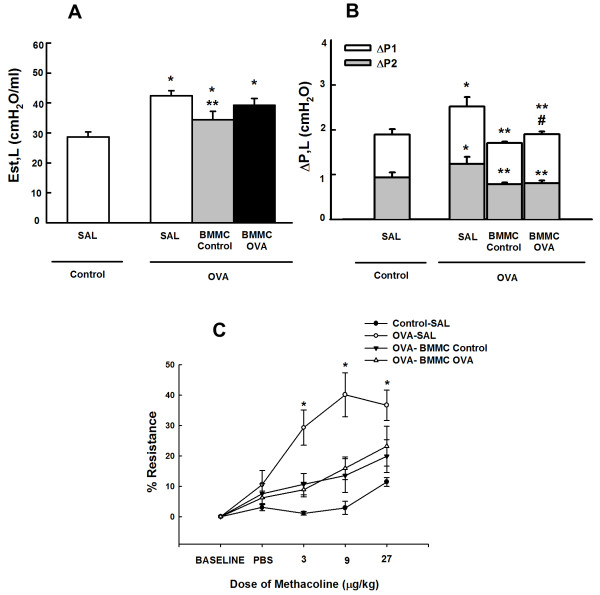
**Lung mechanics. (A)** Lung static elastance (E_st,L_), **(B)** resistive (ΔP1, white bar), viscoelastic (ΔP2, gray bar), and total pressures (ΔPtot = ΔP1 + ΔP2), and **(C)** airway hyperresponsiveness. Control, mice sensitized and challenged with saline; OVA, mice sensitized and challenged with ovalbumin; SAL, mice sensitized and challenged with ovalbumin and treated with saline; BMMC-Control, mice sensitized and challenged with ovalbumin and treated with bone marrow mononuclear cells (BMMCs) obtained from control donors 24 hours after last challenge (2 × 10^6^ cells/mouse); BMMC-OVA, mice sensitized and challenged with ovalbumin and treated with BMMCs obtained from OVA donors 24 hours after last challenge (2 × 10^6^ cells/mouse). *Significantly different from control (*P* < 0.05). **Significantly different from OVA-SAL mice (*P* < 0.05). #Significantly different from BMMC-Control mice (*P* < 0.05). One-way analysis of variance followed by Tukey’s test. PBS, phosphate-buffered saline.

OVA-SAL animals presented higher static lung elastance and resistive (ΔP_1,L_) and viscoelastic (ΔP_2,L_) pressures compared with controls (Figure 3). OVA mice in the BMMC-Control group, but not those in the BMMC-OVA group, demonstrated a significant decrease in static lung elastance (Figure 3A). BMMCs significantly decreased ΔP_1,L_ and ΔP_2,L_ regardless of donor type; however, these decrements in ΔP_1,L_ were statistically more pronounced after administration in BMMC-Control mice compared with BMMC-OVA mice (Figure 3B). Furthermore, the increase in airway resistance induced by methacholine was significantly augmented in the OVA-SAL group. Both the BMMC-Control and BMMC-OVA groups presented comparable decreases in OVA-stimulated increases in airway resistance (Figure 3C).

### BMMC-Control mice showed more effectively reduced lung morphology changes compared with BMMC-OVA mice

**Figure 4:**
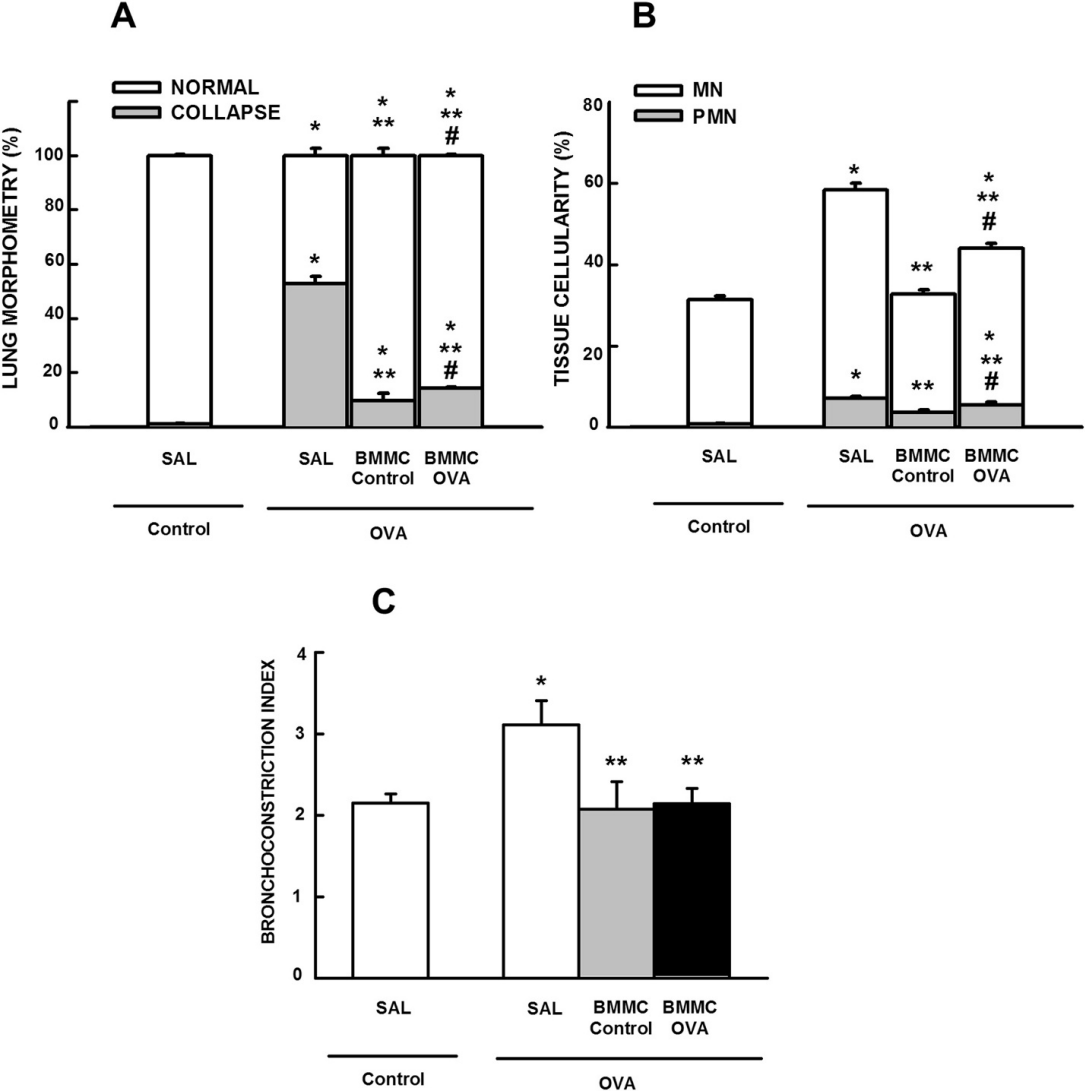
**Morphometric parameters. (A)** Fractional area of normal (white bar) and collapsed alveoli (gray bar), **(B)** mononuclear (MN, white bar) and polymorphonuclear (PMN, gray bar) cells, and **(C)** bronchoconstriction index. All values were computed in 10 random, noncoincident fields of view per mouse. Control, mice sensitized and challenged with saline; OVA, mice sensitized and challenged with ovalbumin; SAL, mice sensitized and challenged with ovalbumin and treated with saline; BMMC-Control, mice sensitized and challenged with ovalbumin and treated with bone marrow mononuclear cells (BMMCs) obtained from control donors, 24 hours after last challenge (2 × 10^6^ cells/mouse); BMMC-OVA, mice sensitized and challenged with ovalbumin and treated with BMMCs obtained from OVA donors, 24 hours after last challenge (2 × 10^6^ cells/mouse). *Significantly different from control (*P* < 0.05). **Significantly different from OVA-SAL mice (*P* < 0.05). #Significantly different from BMMC-Control mice (*P* < 0.05). One-way analysis of variance followed by Tukey’s test.

**Figure 5:**
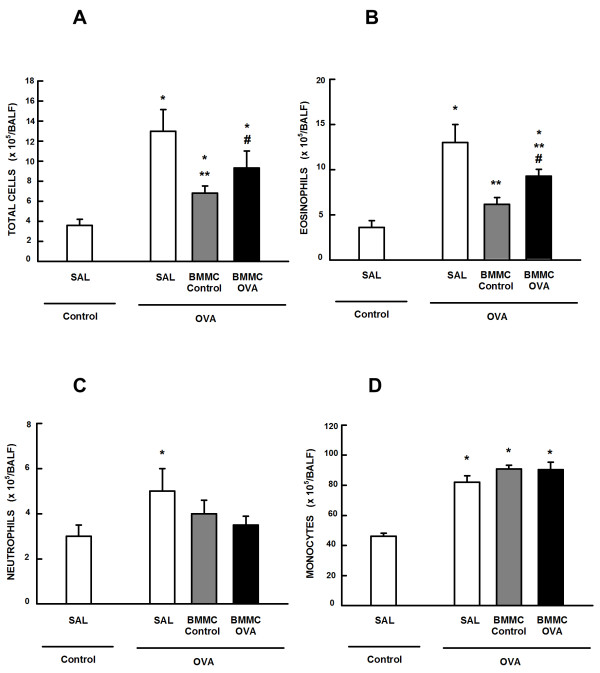
**Total and differential cell counts and protein levels in bronchoalveolar lavage fluid. (A)** Number of total cells, **(B)** eosinophils, **(C)** neutrophils, and **(D)** monocytes in bronchoalveolar lavage fluid (BALF). Control, mice sensitized and challenged with saline; OVA, mice sensitized and challenged with ovalbumin; SAL, mice sensitized and challenged with ovalbumin and treated with saline; BMMC-Control, mice sensitized and challenged with ovalbumin and treated with bone marrow mononuclear cells (BMMCs) obtained from control donors, 24 hours after last challenge (2 × 10^6^ cells/mouse); BMMC-OVA, mice sensitized and challenged with ovalbumin and treated with BMMCs obtained from OVA donors, 24 hours after last challenge (2 × 10^6^ cells/mouse). *Significantly different from control (*P* < 0.05). **Significantly different from OVA-SAL mice (*P* < 0.05). #Significantly different from BMMC-Control mice (*P* < 0.05). One-way analysis of variance followed by Tukey’s test.

The fractional area of alveolar collapse, bronchoconstriction index, and number of total, mononuclear, and polymorphonuclear cells were higher in OVA-SAL mice compared with control animals. These parameters were reduced in BMMC-Control and BMMC-OVA mice, but BMMC-Control mice were more effective at reducing alveolar collapse and number of total, mononuclear and polymorphonuclear cells than BMMC-OVA mice (Figure 4A,B). Both BMMC-Control and BMMC-OVA mice presented a comparable decrease in the OVA-stimulated increase in bronchoconstriction index (Figure 4C).Collagen fiber content in the airways and alveolar septa was significantly increased in the OVA-SAL group as compared with the control group. BMMCs reduced the amount of collagen fibers in alveolar septa regardless of donor type, with a greater reduction in BMMC-Control mice. Neither BMMC-Control nor BMMC-OVA mice showed reduced collagen fiber content in the airways (Figure 5).

### BMMC-Control mice yielded greater reduction in BALF cellularity than BMMC-OVA mice

**Figure 6:**
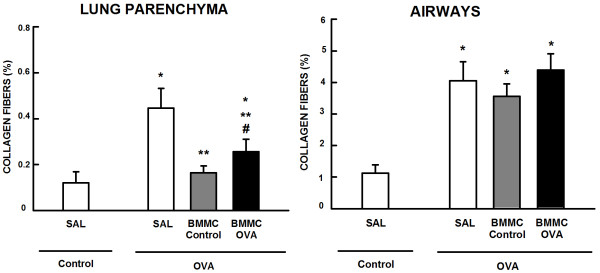
**Collagen fiber content in alveolar septa and airways.** Collagen fiber content in lung parenchyma and airways of mice. All values were computed in 10 random, noncoincident fields of view per mouse. Control, mice sensitized and challenged with saline; OVA, mice sensitized and challenged with ovalbumin; SAL, mice sensitized and challenged with ovalbumin and treated with saline; BMMC-Control, mice sensitized and challenged with ovalbumin and treated with bone marrow mononuclear cells (BMMCs) obtained from control donors, 24 hours after last challenge (2 × 10^6^ cells/mouse); BMMC-OVA, mice sensitized and challenged with ovalbumin and treated with BMMCs obtained from OVA donors, 24 hours after last challenge (2 × 10^6^ cells/mouse). *Significantly different from control (*P* < 0.05). **Significantly different from OVA-SAL mice (*P* < 0.05). #Significantly different from BMMC-Control mice (*P* < 0.05). One-way analysis of variance followed by Tukey’s test.

The number of total cells, monocytes, neutrophils, and eosinophils in BALF was higher in the OVA-SAL group compared with the control group (Figure 6). Both BMMC-Control and BMMC-OVA mice presented statistically significant reductions in total cells, eosinophils, and neutrophils. Notably, reductions in total cells and eosinophils were statistically greater in BMMC-Control mice. No significant changes were observed in BALF monocyte counts, regardless of the BMMC donor group.

### BMMC-Control mice showed greater reductions in cytokine and growth factor levels compared with BMMC-OVA mice

**Figure 7:**
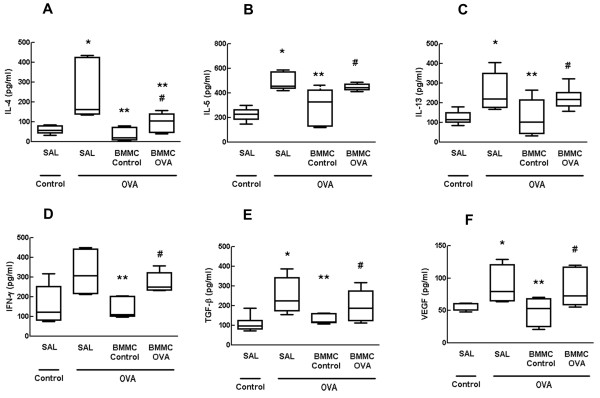
**Cytokines and growth factors in lung tissue.** Levels of **(A)** interleukin (IL)-4, **(B)** IL-5, **(C)** IL-13, **(D)** interferon gamma (IFN-γ), **(E)** transforming growth factor beta (TGF-β), and **(F)** vascular endothelial growth factor (VEGF) in lung tissue. Control, mice sensitized and challenged with saline; OVA, mice sensitized and challenged with ovalbumin; SAL, mice sensitized and challenged with ovalbumin and treated with saline; BMMC-Control, mice sensitized and challenged with ovalbumin and treated with bone marrow mononuclear cells (BMMCs) obtained from control donors, 24 hours after last challenge (2 × 10^6^ cells/mouse); BMMC-OVA, mice sensitized and challenged with ovalbumin and treated with BMMCs obtained from OVA donors, 24 hours after last challenge (2 × 10^6^ cells/mouse). *Significantly different from control (*P* < 0.05). **Significantly different from OVA-SAL mice (*P* < 0.05). #Significantly different from BMMC-Control mice (*P* < 0.05). Kruskal–Wallis test followed by Dunn’s test.

Levels of IL-4, IL-5, IL-13, IFNγ, TGFβ, and VEGF in lung tissue homogenates were higher in the OVA-SAL group than in the control group. Both BMMC-Control and BMMC-OVA mice showed significantly reduced levels of IL-4, IL-5, IL-13, IFNγ, TGFβ, and VEGF; however, these reductions were more pronounced for BMMC-Control mice (Figure 7).

## Discussion

This study investigated the effects of OVA sensitization and challenge, a method commonly used to induce experimental allergic asthma, on the profile of BMMCs. Subsequently, BMMCs from control and OVA donors were administered intratracheally to OVA sensitization and challenge animals. Bone marrow mononuclear fraction of OVA mice exhibited several alterations in its cell composition as compared with bone marrow mononuclear fraction of Control mice, including increases in eosinophil, monocyte, and hematopoietic stem cell populations, as well as a reduction in mesenchymal stem cell populations. Intratracheal administration of both BMMC-OVA and BMMC-Control mice reduced airway responsiveness and the bronchoconstriction index. Nevertheless, BMMC-Control mice presented greater improvement in lung mechanics and histology, and showed more pronounced reductions in collagen fiber deposition in alveolar septa, total number of cells (mainly eosinophils and neutrophils) in BALF, and levels of IL-4, IL-5, IL-13, IFNγ, TGFβ, and VEGF in lung tissue.

The complex interaction between cells present in the bone marrow microenvironment is crucial for providing signals necessary for maintenance of progenitor populations at varying stages of lineage commitment that may differentiate into specific cellular types. These signals include inflammatory cells that may contribute to perpetuation of chronic inflammation in asthma and, conceivably, structural cells that could contribute either to aberrant remodeling or to repair of morphological changes characteristic of asthma, notably subepithelial fibrosis and airway smooth muscle hyperplasia [23]. In the current study, OVA administration induced an increase in hematopoietic precursors that could represent an attempt by the body to provide support for the chronic inflammatory process characteristic of asthma through differentiation of these cells into inflammatory cells, mainly eosinophils, which were increased in the bone marrow of OVA animals. Further studies on lineage commitments will be necessary to address this hypothesis.

Different behaviors of bone marrow cells in the OVA group can be determined by patterns of cytokines and growth factors released during the inflammatory process to which the cells are subjected. Several studies have demonstrated that bone marrow stromal cells and microvascular endothelial cells can respond to OVA inflammatory stimuli by increasing transcription and translation of eosinophilopoietic cytokines, resulting in an increase in eosinophil numbers in the bone marrow microenvironment [24–26]. The commitment of bone marrow progenitors to eosinophils is augmented by an interaction between several proteins stored in the numerous granules contained within the cytoplasm of these leukocytes, such as the secondary granule proteins major basic protein-1 (MBP-1) and eosinophil peroxidase (EPX), and a triad of proinflammatory cytokines commonly found in allergic tissue, including IL-4 and IL-5. A recent study observed that IL-5-induced eosinophilopoiesis was inhibited in knockout MBP-1^–^/EPX^–^ mice [27, 28]. On the other hand, some cytokines are negative regulators of eosinophil differentiation; for example, IL-12 selectively inhibits eosinophilopoiesis from bone marrow stem cells through IFNγ induction [29, 30], reinforcing the importance of the T-helper type 1 versus T-helper type 2 balance in allergic asthma. In the present study, we suggest that the release of T-helper type 2 and T-helper type 1 mediators may have increased the number of eosinophils in bone marrow. No differences were observed in the B-lymphocyte and T-lymphocyte percentages among the bone marrow mononuclear fraction of control and OVA mice, since the differentiation of these cells from naïve lymphocytes generally occurs in peripheral organs, such as the thymus.

Monocytes were increased in BMMC-OVA animals. This increase may be related to the degree of bone marrow stimulation by OVA sensitization and challenge, which results in greater differentiation of hematopoietic stem cells into granulocytes and monocytes due to the T-helper type 2 cytokine profile present in the bone marrow microenvironment [23, 26].

BMMCs also contain MSCs that are associated with important immunomodulatory effects [31, 32]. In this study, the percentage of MSCs was reduced in BMMCs from OVA-induced lung inflammation mice, which may explain the different effects observed with BMMC administration, where BMMC-OVA mice exhibited less immunomodulatory ability to reduce the levels of all cytokines and growth factors analyzed as compared with BMMC-Control mice.

In recent years, a number of experimental studies have demonstrated that systemic MSC administration reduces airways hyperresponsiveness and lung inflammation in mouse models of allergic asthma [5–8]. Postulated mechanisms include induction of T-regulatory cells and a shift from a T-helper type 2 to a T-helper type 1 environment [33, 34]. However, MSCs present potential theoretical disadvantages, including expense and need for culture expansion of non-HLA-matched allogeneic MSCs obtained from other sources. Endogenous MSCs resident in the lung may play a role in asthma pathogenesis, because sensitization and challenge with OVA is accompanied by an increase in lung MSCs that may contribute to fibrotic responses [35]. Based on these considerations, we have evaluated the effects of BMMCs in experimental allergic asthma. These cells can be easily and safely administered on the day of harvesting, express several genes involved in inflammatory response and chemotaxis [12], and present lower cost and increased ease of handling compared with MSCs. Both systemic and intratracheal administration of BMMCs obtained from syngeneic normal mice, when administered before challenge, reduced the inflammatory and remodeling processes, thus improving lung function [9, 10]. Notably, MSCs make up approximately 3% of the BMMC fraction. In comparison with administration of MSCs, BMMCs administered after the last challenge were equally effective as MSCs in reducing airway inflammation, but BMMCs were associated with greater benefit in terms of reducing levels of fibrogenesis-related growth factors. This observation suggests that the MSC fraction of BMMCs may potentiate reduction of lung inflammation. In this line, the more pronounced improvement in lung mechanics observed after BMMC administration suggests that the interaction between multiple cell types present in the BMMCs can play an important role in these processes [11]. BMMCs would therefore arguably be more suitable for clinical use. However, in the clinical setting, bone marrow may be altered by the chronic inflammatory process of asthma. The beneficial effects previously observed after administration of BMMCs from healthy donors may thus not be observed in the case of asthmatic donors.

Based on our previous studies, BMMCs were administered 24 hours after the last challenge, when airway inflammation and remodeling processes are already established [11, 13], thus resembling human asthma. BMMCs were administered intratracheally to achieve a greater number of BMMCs in the site of injury, yielding better effects compared with systemic administration of BMMCs [10, 36].

In this study, administration of BMMCs from OVA and control donors was effective in reducing lung tissue levels of relevant cytokines and growth factors implicated in OVA-induced lung inflammation pathogenesis, which resulted in decreased polymorphonuclear cells as well as eosinophil and neutrophil infiltration in the alveolar septa and BALF, respectively. However, these reductions were more pronounced in BMMC-Control mice compared with BMMC-OVA mice, probably due to the lower percentage of MSCs present in the bone marrow fraction of OVA sensitization and challenge mice, thus resulting in lower immunomodulatory ability [31, 32]. Administration of BMMCs from control donors and, less efficiently, BMMCs from OVA donors therefore modulates steps in the airway remodeling pathway involving IL-4, IL-5, IL-13, eosinophils, TGFβ, and VEGF. In parallel, BMMC-Control and BMMC-OVA mice presented reduced central airway diameter, leading to a decrease in airway hyperresponsiveness in these groups. Nevertheless, BMMCs from control donors were more effective at reducing alveolar collapse and collagen fiber deposition, which probably led to better lung function by reducing lung mechanical parameters (static elastance and viscoelastic and resistive pressures). These results corroborate the findings of a previous study that evaluated the role of therapy with BMMCs from healthy mice, using the same experimental protocol [9, 10].

## Conclusion

In the present study, BMMCs from control and OVA donors were effective in reducing airway inflammation and remodeling, thus improving lung function. However, improvements in lung mechanics and histology were more evident after administration in BMMC-Control mice, suggesting that the alterations promoted in the composition of BMMCs by the inflammatory processes involved in OVA-induced allergic lung inflammation may decrease the efficacy of this approach. To clarify this issue, systematic evaluations of the roles of the different cell types found in BMMC populations should be conducted in future studies. The transfer of autologous BMMC therapy to clinical practice cannot be achieved while these questions remain open. A possible alternative to solve this problem would be to obtain BMMCs from compatible donors found in cell banks coordinated by government or private institutions, as is now done for bone marrow transplantation.

## Abbreviations

BALF: bronchoalveolar lavage fluid
BMMC: bone marrow-derived mononuclear cell
ΔP_2_: _L_: pressure spent by stress relaxation or viscoelasticity
IFNγ: interferon gamma
IL: interleukin
MSC: mesenchymal stem (stromal) cell
OVA: ovalbumin
OVA-SAL: mice sensitized and challenged with ovalbumin and treated with saline
PBS: phosphate-buffered saline
TGFβ: transforming growth factor beta
VEGF: vascular endothelial growth factor.
